# Photoresponse of graphene field-effect-transistor with n-type Si depletion layer gate

**DOI:** 10.1038/s41598-018-22974-7

**Published:** 2018-03-19

**Authors:** Shiho Kobayashi, Yuki Anno, Kuniharu Takei, Takayuki Arie, Seiji Akita

**Affiliations:** Department of Physics and Electronics, Osaka Prefecture University, Sakai, 599-8531 Japan

## Abstract

Graphene/semiconductor Schottky junctions are an emerging field for high-performance optoelectronic devices. This study investigates not only the steady state but also the transient photoresponse of graphene field-effect transistor (G-FET) of which gate bias is applied through the Schottky barrier formed at an n-type Si/graphene interface with a thin oxide layer, where the oxide thickness is sufficiently thin for tunneling of the charge carrier. To analyze the photoresponse, we formulate the charge accumulation process at the n-Si/graphene interface, where the tunneling process through the SiO_x_ layer to graphene occurs along with recombination of the accumulated holes and the electrons in the graphene at the surface states on the SiO_x_ layer. Numerical calculations show good qualitative agreement with the experimentally obtained results for the photoresponse of G-FET.

## Introduction

Graphene is anticipated for use in high-performance electronic devices because of its extraordinary carrier transport property with single-atomic-layer thickness^1^. A graphene/semiconductor heterojunction is an emerging field of recent optoelectronic devices because of their low light absorption of graphene of 2.3%^2^, which is appropriate for high-performance transparent electrodes of optoelectronic devices such as photosensors and photovoltaic applications^3–13^. In such applications, the charge carrier transport through the graphene/semiconductor heterojunction is crucially important to assess the device performance. Generally, a graphene contact to the doped semiconductor substrates is described as a Schottky contact, which is useful in high-performance optoelectronics and photovoltaic devices^4,10,13–21^. In addition, the presence of the thin tunnel barrier at graphene/semiconductor interface modifies the current-voltage characteristics of the junction. In the case of a metal/Si Schottky junction with a 2–10-nm-thick oxide layer at the interface, which is sufficiently thin for tunneling of charge carriers, the open circuit voltage and short circuit current under light irradiation are strongly suppressed because the photogenerated carriers recombine at the interface states in the thin oxide followed by tunneling^22,23^. Photoresponse similar to the ideal Schottky diode is observed only in a reverse bias regime. Similar characteristics were observed for a graphene-Si Schottky contact^11,13–15,18,20^. In this case, both the trap state at the interface and the unique density of states of graphene should be considered for detailed analyses, where the energy position of Fermi level of graphene can be modulated by the field effect through the oxide layer.

The transfer characteristics of graphene field-effect transistor (G-FET) are governed by the induced charge carrier density by application of the gate voltage. For a graphene/doped Si junction with thin oxide layer at reverse bias, the induced charge carrier in graphene should be balanced to that of Si depletion layer induced by the reverse bias corresponding to the gate bias. Under light irradiation, the excess charge carrier generated in the depletion layer by light irradiation is accumulated at the Si/oxide interface because of the presence of the thin oxide layer, which increases the charge carrier density in the graphene and which consequently increases the drain current of the G-FET. This effect can be used to amplify the photoresponse of the graphene/Si Schottky junction, where the photoresponse appears on the drain current of G-FET^11^. Although detailed analysis of current–voltage characteristics of the graphene/Si Schottky junction with thin oxide layer has been conducted^14^, detailed analysis of the G-FET with the Si Schottky junction with the thin oxide gate remains an open subject.

This study investigates not only the steady state but also the transient photoresponse of G-FET of which gate bias is applied through the Schottky barrier formed at the n-type Si/graphene interface with a thin oxide layer, where the oxide thickness is sufficiently thin to tunnel the charge carrier. To analyze the photoresponse, we formulate the charge accumulation process at n-Si/graphene Schottky interface, where the tunneling process through the SiO_x_ layer is considered in addition to the recombination of the accumulated holes and the electrons in the graphene at the surface states on SiO_x_ layer.

## Results and Discussion

**Figure 1:**
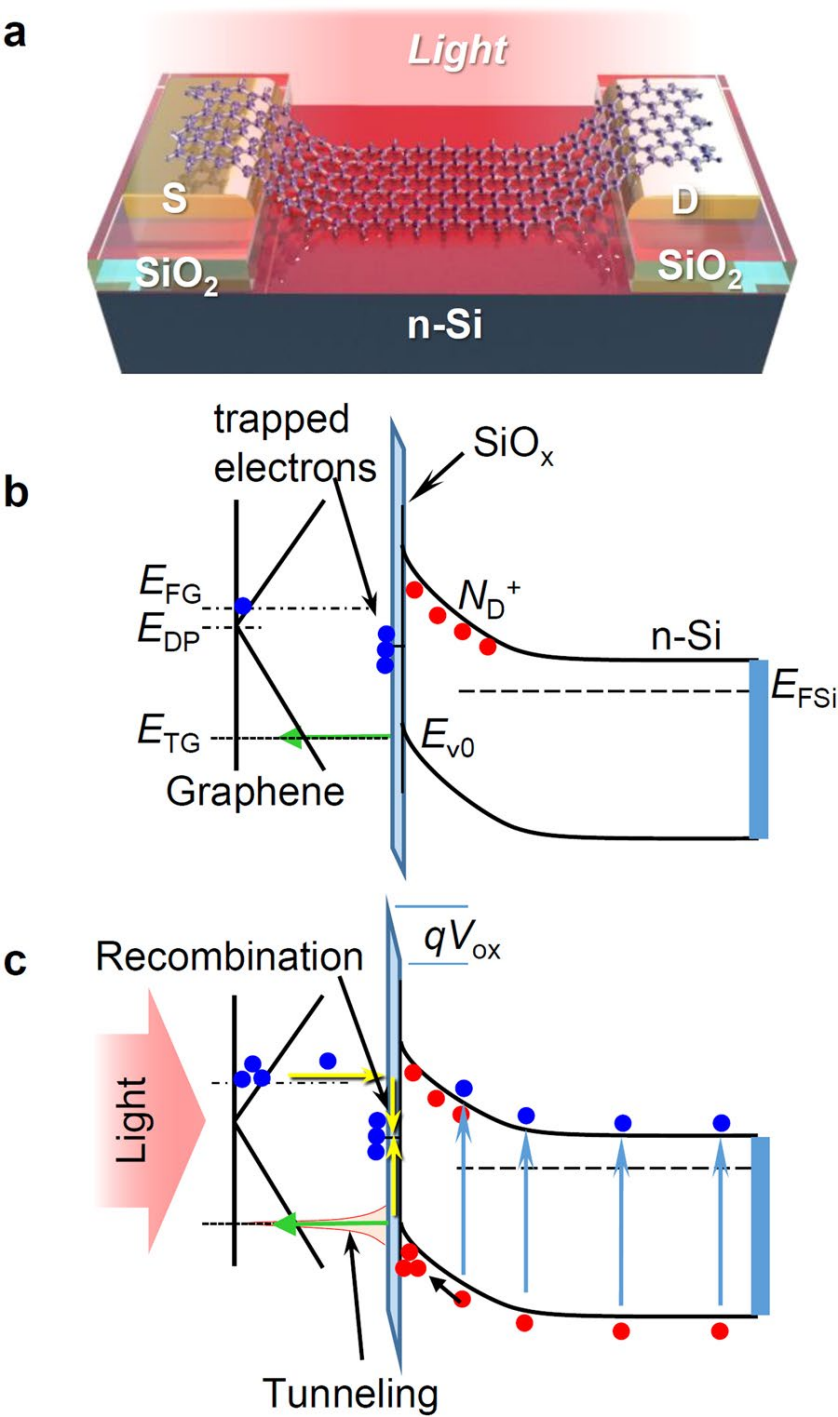
Model of G-FET with Si depletion layer gate. (**a**) Schematic of G-FET with Si depletion layer gate with thin SiO_x_ layer at the interface and energy band profiles at Graphene/n-Si contact under reverse bias (**b**) in a dark condition and (**c**) under light irradiation.

Figure 1a presents a schematic illustration of the G-FET, where the depletion layer formed at the n-Si/graphene Schottky contact with thin SiO_x_ layer acts as the insulating layer of the gate. All processes were performed in air, so that the channel part of the graphene is contacted to the Si surface through a 2–5 nm thick native oxide layer, which is sufficiently thin to tunnel the charge carrier^22,23^. As depicted schematically in Fig. 1b, the Schottky junction is formed at the interface between the Si and graphene, where the depletion layer at Si is developed at the positive gate bias (*V*_GS_) corresponding to the reverse bias of the Si/graphene Schottky junction.

At *V*_GS_ > 0, the photoinduced hole–electron pair at the depletion layer is separated by the build-in potential, as presented schematically in Fig. 1c. For the light source used in this study, we used a light emitting diode (LED) with 623 nm wavelength. The absorption coefficient of Si to 623 nm light is ~3000 cm^−1^, so that the penetration depth of the 623 nm light is ~3 μm, which is much greater than the depletion layer thickness. Consequently, the hole–electron pair is generated throughout the depletion layer. The photo-excited electrons at the depletion region drift to the back gate electrode that is positively biased. The holes at the depletion region also drift toward the graphene channel. Thus, the either direct or trap controlled recombination of photogenerated hole and electron in the depletion region is suppressed. For an ideal Schottky junction, the holes are swept out to the graphene without restriction. However, under the presence of the thin SiO_x_, the holes are accumulated at the interface, followed by tunneling to the graphene, which closely resembles the inversion layer at MOS interface for n-type semiconductor. In this case, the electrons are induced in the graphene as the counter charge of the accumulated holes at the interface, which engenders the Fermi level shift of graphene. Additionally, one must consider recombination of the photogenerated holes and electrons in the graphene through the recombination center at SiO_x_. Consequently, the output current (*I*_DS_) of the G-FET is increased by light irradiation depending on the accumulated holes at the Si/SiO_2_ interface. Similar concept of the trap controlled band modification for the enhancement of photosensitivity has been reported in organic photodiodes^24–28^.

Based on the model described above, we will formulate the light-intensity dependence of *I*_DS_, which is determined by the carrier density of graphene *n*_G_. For simplicity, we assume that the recombination life-times of both of electrons and holes are the same to match *τ*_R_ of the Shockley–Read–Hall (SRH) recombination model^29^. Furthermore, we assume that the intrinsic carrier density is negligibly small. Therefore, the resultant rate equation of accumulated holes, *p*_Si_, at the Si/SiO_2_ interface can be expressed as

1 $$\frac{d{p}_{Si}}{dt}=-\frac{{p}_{Si}}{{\tau }_{T}}-\frac{1}{{\tau }_{R}}\frac{{n}_{G}{p}_{Si}}{{n}_{G}+{p}_{Si}}+g,$$

where the first and second terms at right respectively correspond to the direct tunneling of the accumulated holes into graphene through the thin SiO_x_ and the SRH recombination of the accumulated holes and the electrons in graphene at SiO_x_. Also, *τ*_T_^−1^ stands for the tunneling probability of the accumulated holes, *τ*_R_^−1^ expresses the recombination rate of the accumulated holes and the electrons in graphene at SiO_x_, and *g* denotes the photo-generation rate of holes in the depletion layer. The density of electrons in graphene at Fermi level *E*_FG_ is known to be^1,30–32^.

2 $${n}_{G}={({E}_{FG}-{E}_{DP})}^{2}/\pi {\hslash }^{2}{{v}_{F}}^{2},$$

where *E*_DP_ is the energy position of the charge neutral point, the Dirac point, ${\rm{\hslash }}$ is Planck’s constant, and *v*_F_ represents the Fermi velocity of charge carriers in graphene (~10^8^ cm/s). The tunneling probability *τ*_T_^−1^ of holes into graphene is proportional to the graphene density *D*_G_(*E*_TG_) as final states for tunneling, where *E*_TG_ is the energy position for hole tunneling. The graphene density at energy *E* is

3 $${D}_{G}(E-{E}_{DP})=2(E-{E}_{DP})/\pi {\hslash }^{2}{{v}_{F}}^{2},$$

which is also crucially important for ascertaining *E*_FG_ − *E*_DP_ . At *V*_GS_ higher than that at the gate voltage corresponding to the Dirac point (*V*_Dirac_) under the dark condition, the excess *V*_GS_ (=*V*_GS_ − *V*_Dirac_) is applied for development of the depletion layer, so that one can assume *E*_FG_ ~ *E*_DP_ . At this condition, the energy state for tunneling of accumulated holes into graphene, which corresponds to the energy position of valence band top of Si at the SiO_2_/Si interface, is defined as *E*_v0_. To clarify the tunneling process under light irradiation, we investigate the relation between *E*_TG_ and *E*_v0_, where we consider the Fermi level shift of graphene Δ*E*_FG_ = *E*_FG_ − *E*_DP_ and the additional potential drop at the thin SiO_x_, Δ*V*_OX_, because of *n*_G_ as the counter charge of accumulated holes induced by photoexcitation, *p*_Si_. The relation between *E*_TG_ and *E*_v0_ under the light irradiation is given as

4 $${E}_{TG}-{E}_{DP}={E}_{v0}+q{\rm{\Delta }}{V}_{OX}-{\rm{\Delta }}{E}_{FG}\approx {E}_{v0}+q{\rm{\Delta }}{V}_{OX}-\sqrt{\pi {\hslash }^{2}{{v}_{F}}^{2}{n}_{G}}.$$

Using the combination of Eqs (3) and (4), one can ascertain the density of states of graphene at energy of tunneling hole expressed as

5 $${D}_{G}({E}_{TG}-{E}_{DP})=2{(\pi {\hslash }^{2}{{v}_{F}}^{2})}^{-1}({E}_{v0}+q{\rm{\Delta }}{V}_{OX}-{\rm{\Delta }}{E}_{FG})\approx 2{(\pi {\hslash }^{2}{{v}_{F}}^{2})}^{-1}({E}_{v0}+{q}^{2}{n}_{G}/{C}_{OX}-\sqrt{\pi {\hslash }^{2}{{v}_{F}}^{2}{n}_{G}}),$$

where we assume that *n*_G_ at dark is negligible. In this case, one can expect *n*_G_ ~ *p*_Si_. As described above, tunneling probability *τ*_*Τ*_^−1^ is proportional to the final density of states as

6 $${\tau }_{T}^{-1}\approx \alpha {D}_{G}({E}_{TG})=2\alpha {(\pi {\hslash }^{2}{v}_{F}^{2})}^{-1}({E}_{v0}+{q}^{2}{n}_{G}/{C}_{OX}-\sqrt{\pi {\hslash }^{2}{v}_{F}^{2}{n}_{G}}),\approx 2\alpha {(\pi {\hslash }^{2}{v}_{F}^{2})}^{-1}({E}_{v0}+{q}^{2}p{}_{Si}/{C}_{OX}-\sqrt{\pi {\hslash }^{2}{v}_{F}^{2}{p}_{Si}})$$

where *α* is the constant for proportional factor including the transmittance of tunneling electrons through the thin SiO_2_. Inserting this equation to Eq. (1) with *n*_G_ ~ *p*_Si_ and grouping *p*_Si_ terms, one obtains

7 $$\frac{d{p}_{Si}}{dt}\approx -2\alpha {(\pi {\hslash }^{2}{{v}_{F}}^{2})}^{-1}{p}_{Si}({E}_{v0}+{q}^{2}{p}_{Si}/{C}_{OX}-\sqrt{\pi {\hslash }^{2}{{v}_{F}}^{2}{p}_{Si}})-\frac{{p}_{Si}}{{\tau }_{R}}+g=-{\beta }_{1}{p}_{Si}\,-{\beta }_{2}{{p}_{Si}}^{2}+{\beta }_{3}{{p}_{Si}}^{3/2}+g,$$

where ${\beta }_{1}=2\alpha {(\pi {\hslash }^{2}{{v}_{F}}^{2})}^{-1}{E}_{v0}+{\tau }_{R}^{-1}$, ${\beta }_{2}=2\alpha {(\pi {\hslash }^{2}{{v}_{F}}^{2})}^{-1}{q}^{2}/{C}_{OX}$ and ${\beta }_{3}=2\alpha {(\sqrt{\pi }\hslash {v}_{F})}^{-1}$. At weak light intensity, the contribution of *β*_1_ term is dominant, where the tunneling process and the recombination at SiO_x_ are the main causes. The light intensity (corresponding to *g*) dependence of *p*_Si_ should be expressed as *g*^*θ*^ with *θ* = 1 at the steady state, i.e., *dp*_Si_/*dt* = 0. For the case in which *β*_2_ originating from the voltage across the thin oxide layer is dominant at stronger light intensity, *θ* is approx. 0.5. As presented in Fig. S1 in *Supplementary Information*, the numerically calculated light-intensity dependence of *p*_Si_ shows power law dependence with two slopes. It is noteworthy that *n*_G_ ~ *p*_Si_. Thereby, one can expect that the light-intensity dependence of *I*_*DS*_ shows power law dependence of *g*^*θ*^ with *θ* < 1.

**Figure 2:**
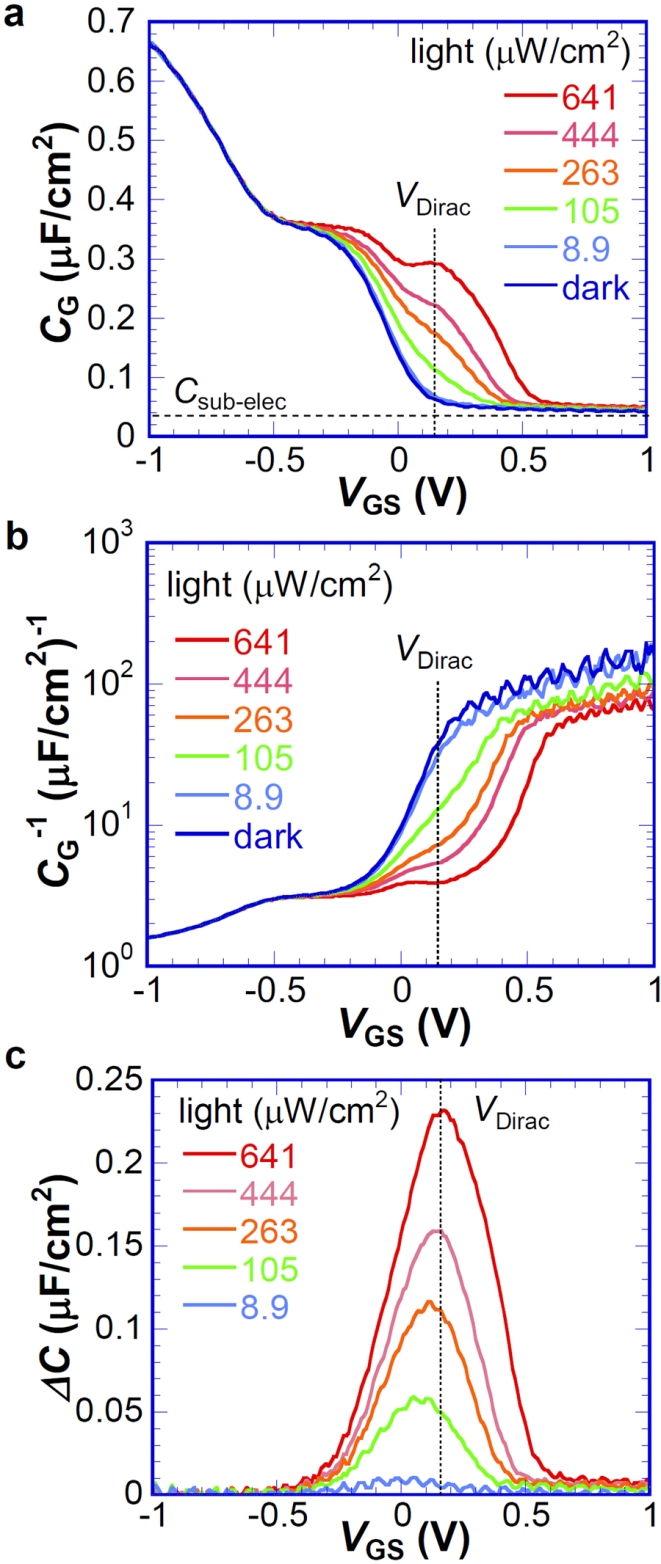
C-V characteristics under light irradiation. (**a**) *V*_GS_ dependence of *C*_G_ under various light intensities, (**b**) *V*_GS_ dependence of *C*_G_^−1^ under various light intensities, and (**c**) *V*_GS_ dependence of Δ*C*_G_ under various light intensities. *V*_Dirac_ in the figure corresponds to the charge neutral point.

To investigate the depletion layer at the junction, the capacitance–voltage (*C*_G_–*V*_GS_) characteristics of the Schottky junction were investigated with or without light irradiation, where the measured capacitance *C*_G_ is a series capacitance consisting of a quantum capacitance of graphene *C*_Q_, the thin native oxide layer *C*_OX_, and the depletion layer at the Si/SiO_x_ interface, *C*_D_. As presented in Fig. 2a and b, at *V*_GS_ < −0.5 V corresponding to the accumulation condition for MOS junction, the gate capacitance *C*_G_ decreases with increasing *V*_GS_ because of the decrease of *C*_Q_^33,34^. The maximum capacitance of *C*_G_^−1^ = *C*_ox_^−1^ + *C*_Q_^−1^ at *V*_GS_ < −1 V is greater than 0.65 μF/cm^2^. The SiO_x_ layer thickness is estimated as 5 nm or less. The corresponding band diagram is portrayed in Fig. S2 in *Supplementary Information*. To clarify the light irradiation effect on *C*_G_, the *C*_G_ difference between the dark and the light conditions, Δ*C*_G_, is shown against *V*_GS_ in Fig. 2c. At *V*_GS_ < −0.5 V, Δ*C*_G_ shows no marked light-intensity dependence.

At *V*_GS_ ~ −0.5 V, the *V*_GS_ dependence of *C*_G_ becomes weaker; *C*_G_ depends slightly on the light intensity. This slight dependence implies that this bias region corresponds to the transition from accumulation to weak depletion of Si^29^ (Fig. S2b to S2c), which results in the appearance of *C*_D_. This *C*_D_ induces the decrease of the applied voltage dependence of the Fermi level shift of the graphene, which gives rise to the weaker *V*_G_ dependence of *C*_Q_. Under light irradiation, the depletion layer thickness decreases because of the photogenerated charge in Si (Fig. S2g), which causes the increase of *C*_D_ and the accumulation of photogenerated holes at the SiO_x_/Si interface, resulting in the increase of voltage applied to SiO_X_: *V*_OX_. Further application of *V*_GS_ increases the depletion region thickness at Si and therefore decreases *C*_G_ in the dark condition in addition to the decrease of *C*_Q_. Under light irradiation, *C*_G_ at −0.5 < *V*_GS_ < 0.5 V increases concomitantly with increasing light intensity, which indicates a decrease of the depletion region by light irradiation. At *V*_GS_ > 0.5 V, *C*_G_ decreases to capacitance originated from the stray capacitance because the electrode pads were connected in parallel.

**Figure 3:**
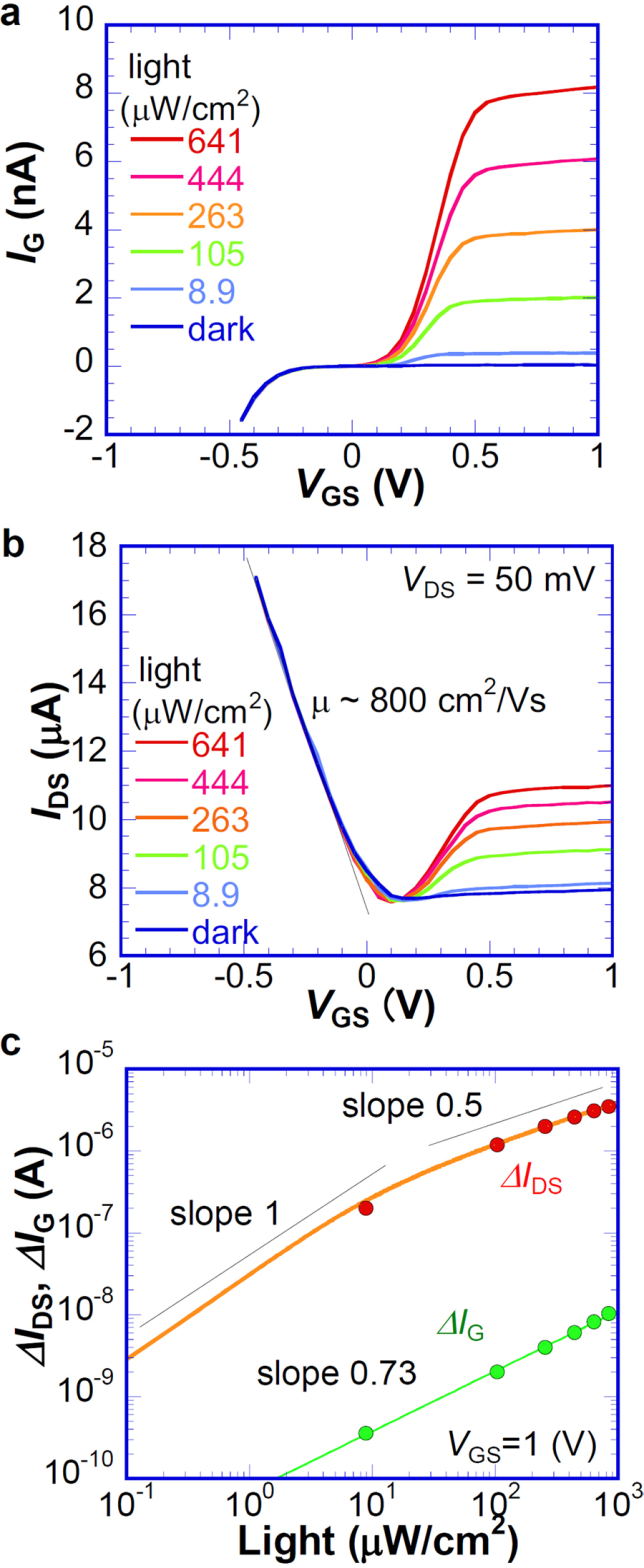
Steady state electrical characteristics under light irradiation. (**a**) *V*_GS_ dependence of *I*_G_ corresponding to the current passing through the graphene/n-Si junction under various light intensities. (**b**) *V*_GS_ dependence of *I*_DS_ under various light intensities measured at *V*_DS_ = 50 mV. (**c**) Light-intensity dependence of Δ*I*_G_ and Δ*I*_DS_ at *V*_DS_ = 50 mV and *V*_G_ = 1 V. The solid curve shown in the figure is the numerically calculated curve Δ*I*_DS_ shown in Fig. S1.

Figure 3a presents the gate bias voltage *V*_GS_ dependence of the current passing through the Schottky junction *I*_G_ equivalent to the gate leak current of G-FET under conditions of various light intensities from dark condition to 641 μW/cm^2^, where both the source and drain of the G-FET were connected to ground. At *V*_GS_ > 0, corresponding to reverse bias for the Schottky junction, *I*_G_ in the dark is well blocked (<0.1 nA) by the Schottky barrier. Under the light irradiation, *I*_G_ increases concomitantly with increasing light intensity to 63 nA at 641 μW/cm^2^. In this region, the photo-excited electrons are swept out through the depletion layer of n-Si, whereas the photo-excited holes are tunneled to the graphene through SiO_x_ layer or recombined at the graphene-Si interface at the thin SiO_x_ layer, as presented in Fig. 1c. *I*_G_ at −0.5 V < *V*_GS_ < 0.2 V under light illumination is suppressed, unlike the common Schottky junction behavior. This behavior is commonly observed at the Schottky junction of lightly doped n-Si with thin SiO_x_ layer^22,23^. It originated mainly from the presence of thin SiO_x_ layer at the interface, resulting in the increase of the direct carrier recombination of the photogenerated carriers at the depletion layer of n-Si^14^.

The transfer characteristics of the G-FET under an illumination condition are presented in Fig. 3b, which is very similar to the case for conventional G-FETs^1^ at *V*_G_ < *V*_Dirac_. The drain current *I*_DS_ at *V*_G_ > *V*_Dirac_ in a dark condition remains almost constant, which implies that *E*_FG_ ~ *E*_DP_, as assumed in the numerical calculations. Under light irradiation, *I*_DS_ increases concomitantly with increasing light intensity of 8–11 μA with saturation behavior at higher *V*_GS_, where *V*_Dirac_ (*V*_GS_ ~ 0.15 V) is the bias voltage for the charge neutrality point of G-FET determined at minimum *I*_DS_. The *I*_DS_ difference between the dark-current and photo-current, Δ*I*_DS_, is 3 μA at 641 μW/cm^2^, which is 2–3 orders of magnitude larger than that of Δ*I*_G_ depicted in Fig. 3a, indicating that the direct contribution of gate leak current to *I*_DS_ is negligible.

Based on the band model depicted in Fig. 1b and c, *I*_DS_ is governed by the accumulated holes at SiO_x_/Si interface, even under light irradiation, as described earlier. The ionized donor (positively charged) in the depletion layer gives limited impact to *I*_DS_ at *V*_GS_ > *V*_Dirac_ under light irradiation because of the slight increase of *I*_DS_ in dark conditions. The light-intensity dependence of the saturated *I*_DS_ under light irradiation at *V*_G_ > *V*_Dirac_ indicates that the charge carrier density in graphene is determined by the accumulated photo-excited excess hole at the interface, where the density of the accumulated photo-excited excess hole is regulated by the recombination rate of excess holes through the thin SiO_x_ barrier, as described above. The field effect mobility of hole in graphene was estimated as 800 cm^2^V^−1^s^−1^ from *C*_G_ of around *V*_GS_ = −0.5 V.

Figure 3c presents the light-intensity dependences of Δ*I*_G_ and Δ*I*_DS_, both showing power law dependence of the light intensity. The light-intensity dependence of Δ*I*_G_ can be fitted by one slope of 0.73. By contrast, the light-intensity dependence of Δ*I*_DS_ comprises two slopes as predicted theoretically earlier. The experimental data are well fitted to the numerically calculated result shown in Fig. S1, as indicated by the solid curve in Fig. 3c. For the numerical calculations, *v*_F_ = 0.8 × 10^6^ m/s, *τ*_R_ = 18 ms, *E*_vo_ = 0.05 eV, *α* = 2 × 10^−34^, SiO_x_ thickness = 5 nm are used. The actual *p*_si_, which is proportional to Δ*I*_DS_, is unknown. For that reason, the vertical axis was adjusted with an arbitrary ratio of Δ*I*_DS_*/p*_si_. From a practical perspective for the photosensor, the Δ*I*_DS_ is 2–3 orders of magnitude larger than Δ*I*_G_. This photocurrent amplification of G-FET with the Si Schottky gate gives rise to easier measurement of the light intensity than the simple graphene/Si Schottky diode, where the noise equivalent power (NEP) of the G-FET obtained at 8.9 μW/cm^2^ is 7 × 10^−14^ W/Hz^1/2^ (corresponding specific detectivity: 1 × 10^11^ Jones).

**Figure 4:**
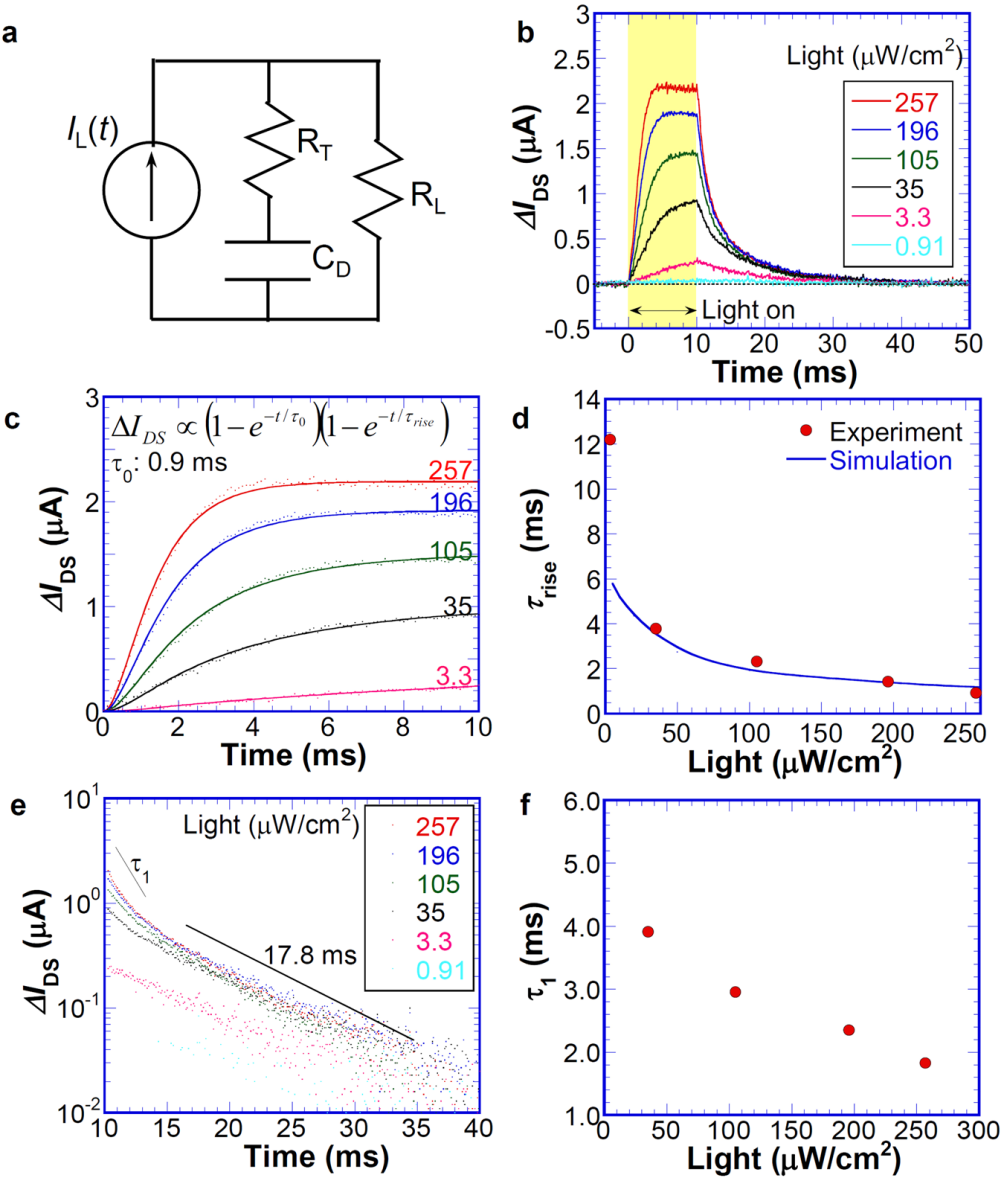
Transient photoresponse. (**a**) Equivalent circuit of the Schottky photodiode. (**b**) Typical transient photoresponse Δ*I*_DS_ of G-FET with n-type Si depletion layer gate under various light intensities at *V*_DS_ = 50 mV and *V*_G_ = 1 V. (**c**) Transient response of Δ*I*_DS_ at light-on under various light intensities. Solid lines are fitting curves. (**d**) Light-intensity dependence of the time constant *τ*_rise_ obtained from the fitting for light-on. (**e**) Semi-log plot of transient response of Δ*I*_DS_ at light-off with various light intensities. (**f**) Light-intensity dependence of the time constant *τ*_1_ for light-off.

Transient response of G-FET is important for practical applications. The photoresponse time of the Schottky diode is usually explained by the simplified model with junction capacitance *C*_D_, internal series resistance *R*_T_, and load resistance *R*_L_, as portrayed schematically in Fig. 4a, where the photo-generated current *I*_L_ is a current source determined by the light intensity. In our device structure, *R*_L_ and *R*_T_ consist of contact resistance, tunnel resistance, and resistance of the Si substrate. Based on the simplified model presented here, the time constant for rise time, *τ*_0_, is defined simply by *C*_D_*R*_T_, expressed as ${I}_{L}[1-\exp (-t/{\tau }_{0})]$. In addition to this time constant, one must consider the time for hole accumulation at the Si/SiO_2_ interface, which induces the time dependence of *I*_L_ determined by Eq. 7. Figure S3 presents the transient response of numerically calculated *p*_Si_ corresponding to *I*_L_ under various light intensities, where the used parameters are the same for those of light-intensity dependence in a steady state. The calculated data are well fitted by $1-\exp (-t/{\tau }_{rise})$ with a different time constant, *τ*_rise_, which depends on the light intensity. Consequently, one can expect that the transient photoresponse at light-on is given as the product of $[1-\exp (-t/{\tau }_{rise})][1-\exp (-t/{\tau }_{0})]$.

Figure 4b presents the transient photoresponse of *I*_DS_ on the G-FET, where the light intensity was varied from 0.91 to 257 μW/cm^2^. As might be readily apparent, the response time at light-on *τ*_rise_ decreases concomitantly with increasing light intensity, which is consistent with the calculated results based on the proposed model shown in Fig. S3, where the higher light intensity causes the faster charge accumulation at the interface. To investigate the transience time, we fitted the transient response at light-on with the function expressed as $[1-\exp (-t/{\tau }_{rise})]$ $[1-\exp (-t/{\tau }_{0})]$. As portrayed in Fig. 4c, the experimental data are well fitted as we expected, where *τ*_0_ determined from *C*_D_*R*_T_ was fixed. Note that the lowest photoresponse obtained at 0.91 μW/cm^2^ is quite noisy and close to the dark signal, so that we could not obtain the reliable fitting curve. The experimentally obtained results were not fitted well by the simple exponential function. The time constant *τ*_rise_ obtained from the fitting clearly depends on the light intensity: it decreases from 12.5 to 1.6 ms with increasing light intensity, as portrayed in Fig. 4d. The solid line represents *τ*_rise_ obtained from the numerically calculated results presented in Fig. S3b, which well reproduces the experimentally obtained results indicating that the hole accumulation process at the interface accelerates the photoresponse time at higher light intensity, as we proposed. Note that the low power region around 3.3 μW/cm^2^ could not be well reproduced based on our simplified model. This is most likely that the contribution of recombination process becomes more dominant at low carrier density. This is a subject for further study.

The transient responses for the light-off condition were described by a double exponential function, as portrayed in Fig. 4e. At *t* > 3 ms, the decay can be expressed by *τ*_f_ = 17.8 ms for all light intensities, mainly because of the recombination time constant *τ*_R_ (see Eqs 1 and 7) for excess holes at the interface. For the numerical calculations presented above, *τ*_R_ = 18 ms was used. At *t* < 3 ms, the time constant *τ*_1_ at this region depends on the light intensity, as depicted in Fig. 4f. Time constant *τ*_1_ closely resembles *τ*_rise_, as portrayed in Fig. 4d, which implies that the tunneling process of accumulated holes at the interface mainly contributes to the high decay rate. Results show that the proposed analysis can explain the experimentally obtained results qualitatively.

## Conclusions

We demonstrated a photosensor consisting of G-FET with gate bias applied through the Schottky barrier formed at an n-Si/graphene interface, where a thin SiO_x_ barrier of ~5 nm is present at the interface. The photocurrent amplification of G-FET with the Si Schottky gate gives rise to easier measurement of light intensity than the simple graphene/Si Schottky diode, where the noise equivalent power (NEP) of the G-FET obtained at 8.9 μW/cm^2^ is 7 × 10^−14^ W/Hz^1/2^. This amplification function is useful for practical applications. To analyze the photoresponse, we formulated the charge accumulation process at the n-Si/graphene interface, where the tunneling process through the SiO_x_ layer was considered in addition to the recombination of the accumulated holes and the electrons in the graphene at the surface states on SiO_x_ layer. The accumulated holes induce the Fermi level shift of graphene, which produces variation of the tunneling probability. The analytical framework proposed here is well described by the experimentally obtained results for both steady states and transient states. Moreover, it provides an effective means of analyzing charge carrier transport through the grapheme-based heterostructure through a thin tunneling barrier, which represents one path to achieving ultrathin opto-electronic devices in the future.

## Methods

The device fabrication process was the following. First, metal electrodes consisting of Cr/Au (5 nm/30 nm) as a source and drain electrodes were fabricated on a Si substrate with a 300 nm-thick SiO_2_ layer using conventional photolithography processing. The resistivity of the n-type Si substrate used in this experiment is 8 Ωcm. Unlike common back-gate G-FETs, we removed the SiO_2_ layer (300 nm thick) between the electrodes using buffered HF. All processes were performed in air, so that the channel part of the graphene is contacted to the Si surface through a 2–5 nm thick native oxide layer. Subsequently, after a monolayer graphene was transferred onto the substrate using polymethyl methacrylate, it was trimmed using oxygen plasma etching to form the G-FET channel, where the graphene was synthesized using low-pressure chemical vapor deposition at 1000 °C using Cu foil as catalyst^35,36^. The channel length and width are, respectively, 70 and 100 μm.

## Electronic supplementary material

Supplementary Information
