# Supplementary material for: Photoresponse of graphene field-effect-transistor with n-type Si depletion layer gate
Source: Sci Rep. 2018 Mar 19;8:4811. doi: 10.1038/s41598-018-22974-7 (PMC5859296; doi:10.1038/s41598-018-22974-7)
Supplement: Supplementary file 1 — Supplementary Information [file 41598_2018_22974_MOESM1_ESM.pdf]

Supplementary information

Photoresponse of graphene field-effect-transistor with n-type Si depletion layer gate

Shiho Kobayashi, Yuki Anno, Kuniharu Takei, Takayuki Arie, Seiji Akita*

Department of Physics and Electronics, Osaka Prefecture University, Sakai 599-8531, Japan

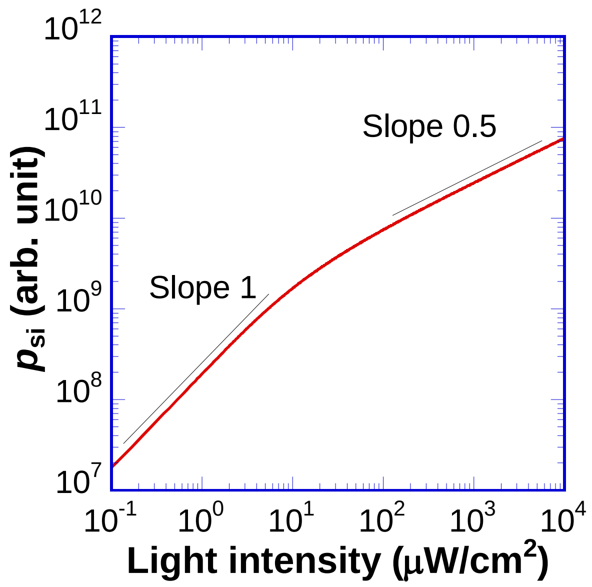

Figure S1. Calculated light intensity dependence of accumulated hole density. Log–log plot of light intensity dependence of p_{Si} at steady state calculated using Eq. 6 in the main text, where $v_F = 0.8\times10^6$ m/s, τ_R = 18 ms, E_{vo} = 0.05 eV, $\alpha = 2\times10^{-34}$, SiO_x thickness of 5 nm is used for numerical calculations.

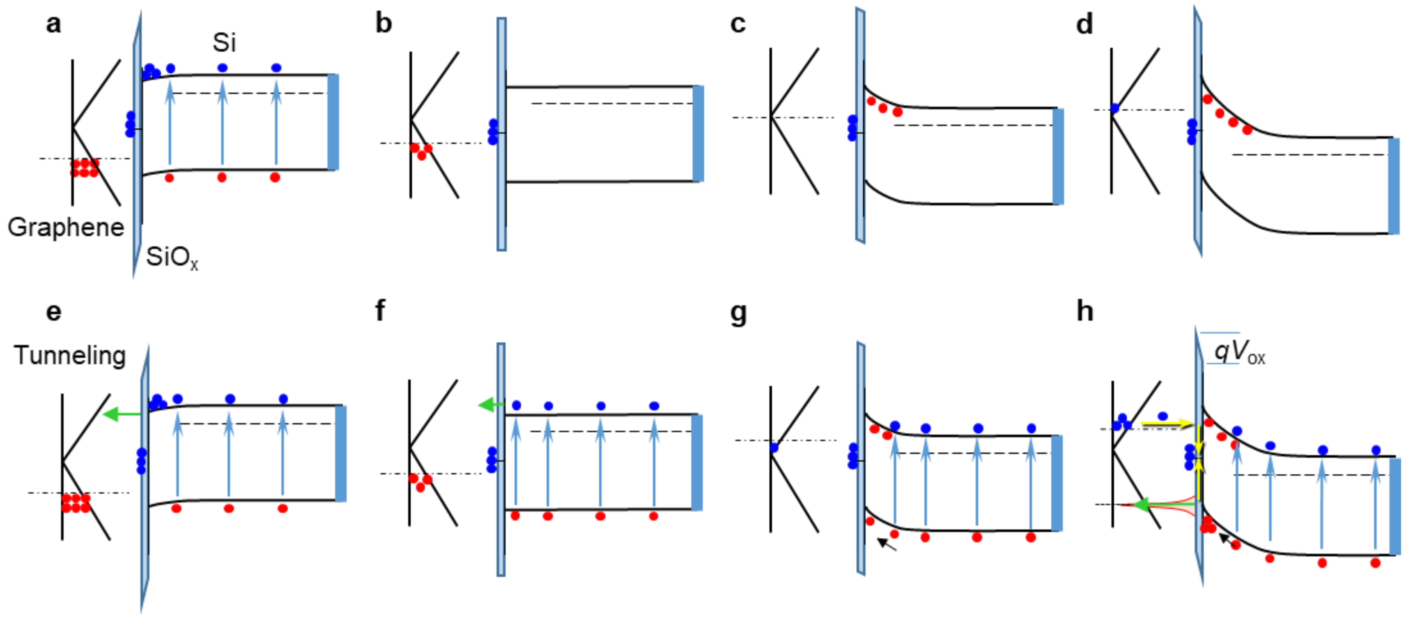

Figure S2 Band diagram for G-FET/n-Si interface with thin SiO$_x$. (a–d) Band diagram at various gate voltages in a dark condition. (e–h) Band diagram at various gate voltages under light irradiation. Blue and red solid circle in the figures respectively denote electrons and holes.

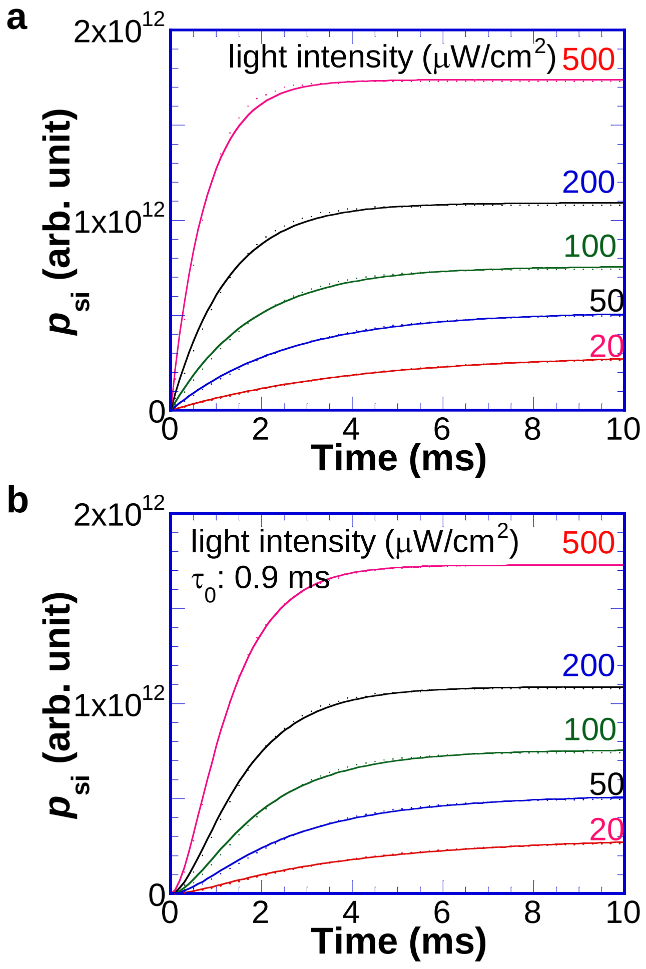

Figure S3 Calculated photoresponse of accumulated holes at SiO_x/Si interface under various light intensities. (a and b) Calculated photoresponses of accumulated holes with and without consideration of the effect of the CR time response τ_0. Solid curves are fitting curves based on (a) the simple exponential function expressed as $1-\exp(-t/\tau_{rise})$ and (b) the combined exponential function expressed as $[1-\exp(-t/\tau_0)][1-\exp(-t/\tau_{rise})]$.
